# Safety of elective abdominal and vascular surgery during the COVID-19 pandemic: a retrospective single-center study

**DOI:** 10.1186/s40001-021-00583-x

**Published:** 2021-09-23

**Authors:** Sven Flemming, Mohammed K. Hankir, Simon Kusan, Manuel Krone, Friedrich Anger, Christoph-Thomas Germer, Armin Wiegering

**Affiliations:** 1 Department of General, Visceral, Transplantation, Vascular and Pediatric Surgery, University Hospital Wuerzburg, Oberduerrbacher Str. 6, 97080 Wuerzburg, Germany; 2 Institute of Hygiene and Microbiology, University of Wuerzburg, Wuerzburg, Germany

**Keywords:** SARS-CoV-2, COVID-19, Elective surgery, Screening, PCR

## Abstract

**Background:**

Patients with coronavirus disease 2019 (COVID-19) who undergo surgery have impaired postoperative outcomes and increased mortality. Consequently, elective and semi-urgent operations on the increasing number of patients severely affected by COVID-19 have been indefinitely postponed.in many countries with unclear implications on disease progression and overall survival. The purpose of this study was to evaluate whether the establishment of a standardized screening program for acute respiratory syndrome coronavirus 2 (SARS-CoV-2) is sufficient to ensure high-quality medical and surgical treatment of COVID-19 and non-COVID-19 patients while minimizing in-hospital SARS-CoV-2 transmission.

**Methods:**

The screening program comprised polymerase chain reaction (PCR) testing of nasopharyngeal swabs and a standardized questionnaire about potential symptoms for SARS-CoV-2 infection. All elective and emergency patients admitted to the surgical department of a tertiary-care hospital center in Lower Franconia, Germany, between March and May 2020 were included and their characteristics were recorded.

**Results:**

Out of the study population (n = 657), 509 patients (77.5%) had at least one risk factor for a potentially severe course of COVID-19 and 164 patients (25%) were active smokers. The average 7-day incidence in Lower Franconia was 24.0/100,000 during the observation period. Preoperative PCR testing revealed four asymptomatic positive patients out of the 657 tested patients. No postoperative SARS-CoV-2 infection or transmission could be detected.

**Conclusion:**

The implementation of a standardized preoperative screening program to both COVID-19 and non-COVID-19 patients can ensure high-quality surgical care while minimizing infection risk for healthcare workers and potential in-hospital transmission.

## Introduction

In response to the COVID-19 pandemic caused by the severe acute respiratory syndrome coronavirus 2 (SARS-CoV-2), many surgical departments have had to indefinitely postpone semi-urgent and elective operations to accommodate the rising number of severely affected patients [1, 2]. This has in turn heightened the risk of in-house SARS-CoV-2 transmission between patients and surgical staff [3–7]. Further, patients with perioperative SARS-CoV-2 infection have increased mortality [8–10]. Since the middle- to long-term consequences of postponing surgery in non-COVID-19 patients are presently unclear, specific containment and testing strategies are mandatory to ensure universally high-quality medical and surgical treatment while minimizing the risk of in-hospital-acquired infections [1, 11].

Presented with this problem, we introduced a number of precautionary measures including standardized preoperative SARS-CoV-2 testing, establishment of COVID-19 and non-COVID-19 areas (intensive care units, non-intensive care units, emergency rooms and operation rooms) and prioritization of operational interventions based on medical urgency and logistical resources (intensive care unit capacity for COVID-19 and non-COVID-19 patients, personnel and material capacities) [1]. The aim of this study was to show that by implementing these precautionary measures, emergency and elective surgeries are both feasible at a tertiary-care hospital center (university hospital) during the COVID-19 pandemic without increasing the risk for nosocomial transmission of SARS-CoV-2.

## Methods

### Study population

**Fig. 1:**
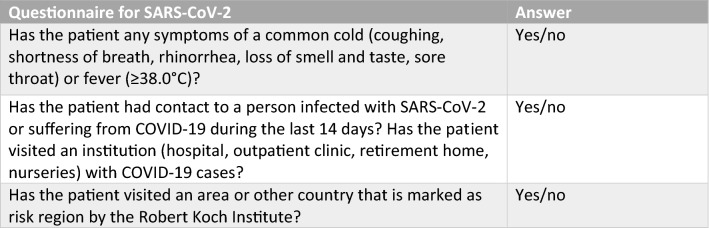
Standardized questionnaire for SARS-CoV-2 infection

Patients admitted to the Department of Surgery I at the University Hospital Wuerzburg between March 26th and May 24th, 2020, had to answer a standardized COVID-19 questionnaire and were tested for SARS-CoV-2 infection by real-time reverse transcriptase polymerase chain reaction (RT-PCR) [12]. The standardized questionnaire enquired about clinical symptoms that are characteristic of symptomatic SARS-CoV-2 infection including coughing, shortness of breath, rhinorrhea, loss of smell and taste, sore throat, fever or diarrhea [13] as well as any potential contact the patient had with suspected or confirmed COVID-19 patients (Fig. 1). A nasopharyngeal swab for RT-PCR testing [14] was performed at our outpatient clinic by specially trained nursing staff within 48 h of elective surgery. In case of emergency, the questionnaire and PCR testing were conducted immediately prior to the operation. In such cases, anesthesia was performed by using personal protection equipment as generally recommended [1, 15]. Based on the outcome of the questionnaire, operations were performed in COVID-19 or non-COVID-19 operation areas.

Demographic variables included age, sex, body mass index (BMI), immunosuppression, and cardio-pulmonary risk factors. Operative variables included urgency of surgery, diagnosis, and surgical procedure. In parallel, the incidence of COVID-19 patients in the district of Lower Franconia was registered on a daily basis [16].

### Statistical analysis

Descriptive data are presented as median with range, mean with standard deviation (SD) or total numbers with percentage. Statistical analysis was performed using SPSS statistics (Version 25, IBM, Armonk, NY, USA).

## Results

During the observation period, 657 patients were admitted to our department with an average age of 59.93 ± 17.92 years and BMI of 26.82 ± 5.39 kg/m^2^, of whom 101 patients underwent emergency surgery and 61 patients received urgent non-surgical treatment. One hundred and sixty-six patients were treated due to a cancer-related problem (Table 1). Almost 12% of patients (n = 78) had a medical history of pulmonary disease including bronchial asthma and chronic obstructive pulmonary disease (COPD), 25% (n = 164) were active smokers and more than three-quarters (77.5%, n = 509) had at least one risk factor for a potentially severe course of COVID-19 [17]. Operative procedures are listed in detail in Table 2, and were performed either using an open, laparoscopic or robotic-assisted approach. Vascular surgery included open and endovascular procedures.

**Table 1:** Baseline characteristics

Variables	
Age (year)	
Mean (SD)	59.93 (17.92)
Median (range)	62 (0–91)
Sex (n, %)	
Female	302 (45.96)
Male	355 (54.04)
Hypertonus (n, %)	377 (57.4)
Diabetes (n, %)	137 (20.9)
Coronary heart disease/peripheral artery occlusive disease (n, %)	169 (25.7)
COPD/asthma (n, %)	78 (11.9)
Immunosuppression (n, %)	81 (12.3)
Active smoking (n, %)	164 (25.0)
Risk factors (n, %)	
= 1	509 (77.5)
> 1	360 (54.8)
> 2	147 (22.4)
> 3	45 (6.8)
Urgency (n, %)	
Emergency	164 (25.0)
Elective	493 (75.0)
Non-cancer/cancer (n, %)	490 (74.6)/167 (25.4)
Treatment procedure (n, %)	
Operation	514 (78.2)
Non-surgical treatment	143 (21.8)

Risk factors include hypertonus, diabetes, coronary heart disease/peripheral artery occlusive disease, COPD/asthma, and immunosuppression

*COPD* chronic obstructive pulmonal disease, *SD* standard deviation

**Table 2:** Type of surgical procedures

Type of operation	All (n, %)	Emergency (n, %)
Appendectomy	13 (2.0)	12 (7.3)
Colostomy formation	1 (0.2)	–
Colostomy reversal	3 (0.5)	–
Diagnostic laparoscopy	18 (2.7)	2 (1.2)
Diagnostic laparotomy	21 (3.2)	14 (8.5)
Drainage of hematoma	4 (0.6)	4 (2.4)
Feeding gastrostomy	1 (0.2)	–
Ileostomy formation	3 (0.5)	–
Ileostomy reversal	1 (0.2)	–
Laparoscopic hernia repair	7 (1.1)	2 (1.2)
Open hernia repair	21 (3.2)	3 (1.8)
Left hemicolectomy	19 (2.9)	4 (2.4)
Right hemicolectomy	8 (1.2)	2 (1.2)
Low anterior rectum resection	10 (1.5)	–
Ileocecal resection	6 (0.9)	–
Small bowel resection	1 (0.2)	–
Esophagectomy	11 (1.7)	2 (1.2)
Esophageal procedure	18 (2.7)	–
Repair of ulcer	3 (0.5)	3 (1.8)
Laparoscopic fundoplication	10 (1.5)	–
Subtotal colectomy	3 (0.5)	–
Total colectomy	4 (0.6)	–
Gastrectomy	11 (1.7)	–
Abscess drainage	12 (1.8)	8 (4.9)
Perineal abscess drainage	11 (1.7)	8 (4.9)
Wound exploration/revision	18 (2.7)	7 (4.3)
Thyroidectomy	38 (5.8)	–
Neck dissection	1 (0.2)	–
Cholecystectomy	26 (4.0)	4 (2.4)
Partial pancreatectomy	10 (1.5)	–
Total pancreatectomy	2 (0.3)	1 (0.6)
Resection of liver segment	6 (0.9)	–
Hemi-hepatectomy	8 (1.2)	–
Other hepato-pancreato-biliary procedure/operation	2 (0.3)	–
Lymph node dissection	3 (0.5)	–
Central venous catheter implantation	34 (5.2)	1 (0.6)
Multi visceral resection	6 (0.9)	–
Femoral artery bypass	15 (2.3)	6 (3.7)
Embolectomy	2 (0.3)	2 (1.2)
Femoral arterial endarterectomy	10 (1.5)	1 (0.6)
Arterio-venous fistula formation	10 (1.5)	5 (3.0)
Abdominal aorta repair	10 (1.5)	2 (1.2)
Femoral artery aneurysm repair	3 (0.5)	1 (0.6)
Percutaneous transluminal angioplasty	53 (8.1)	4 (2.4)
Limb amputation	16 (2.4)	1 (0.6)
Carotid endarterectomy	8 (1.2)	–
Splenectomy	2 (0.3)	1 (0.6)
Adrenalectomy	7 (1.1)	–
Transplantation	1 (0.2)	1 (0.6)

**Fig. 2:**
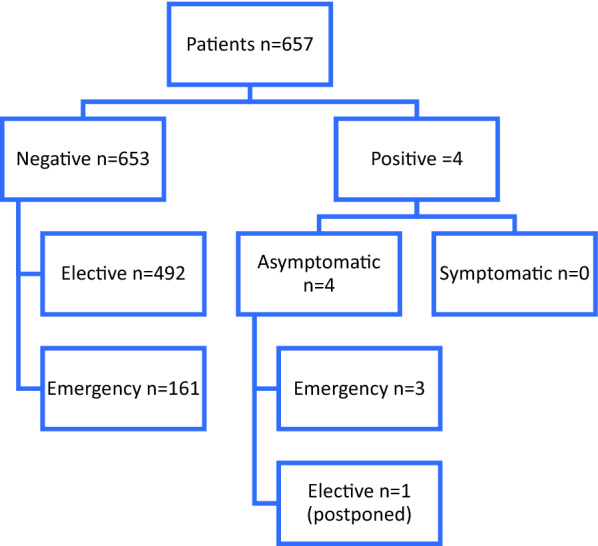
SARS-CoV-2 testing by RT-PCR

The average 7-day incidence in Lower Franconia (1,317,000 residents) was 24.0/100,000 during the observation period [16]. Standardized RT-PCR testing of admitted patients revealed 4 SARS-CoV-2 positive cases—all of whom were asymptomatic. Treatment was delayed in one of these four patients. The other three patients were isolated and treated in dedicated COVID-19 areas (Fig. 2).

During the observation period, an average of 22 patients were hospitalized due to SARS-CoV-2 infection with a maximum of 22 patients in an ICU ward and 16 patients in a non-ICU ward on April 17th. Postoperative viral transmission and SARS-CoV-2 infection, respectively, were not detected and, thus, no COVID-19-related mortality occurred.

## Discussion

Recently published studies showing higher mortality rates after surgery in COVID-19 patients have concluded that the threshold for surgery during the COVID-19 pandemic should be higher [8–10]. This has had the implication that surgical procedures should be postponed and that non-operative therapies are promoted instead [8–10]. However, closer inspection of the data reveals that only 280 of the 1128 patients included in the study by D. Nepogodiev et al. [8] had an elective operation, of whom 250 had postoperative SARS-CoV-2 infection. Thus, in-hospital transmission of SARS-CoV-2 might be possible, since these data were collected during the beginning of the pandemic when health care systems were overwhelmed and there was a lack of sufficient personal protective equipment and test capacities. Given that asymptomatic undetected SARS-CoV-2-positive patients are a potential source of nosocomial transmission, effective screening and containment measures are mandatory to minimize the risk of in-hospital transmission of SARS-CoV-2. Our data show that under COVID-19 pandemic conditions, use of a standardized questionnaire and systematic RT-PCR testing are highly effective tools to identify preoperatively asymptomatic SARS-CoV-2 patients, and thus safely enable elective surgery. While our findings are supported by other studies [18–20], an international survey has shown that standardized screening programs are not yet established in surgical practice [21]. In our test strategy, we excluded the routine computed tomography (CT) of the chest, since its value has been questioned [20, 22], and may thus introduce unnecessary exposure to radiation.

It is well known that cardiovascular comorbidities and cancer are risk factors for a severe disease course of COVID-19 [17]. However, as shown by our analysis and other studies, the introduction of standardized screening programs and the establishment of dedicated COVID-19-free surgical pathways enable elective surgery in these high-risk patients during the COVID-19 pandemic without increasing the risk of postoperative SARS-CoV-2 infection and mortality [23–25].

Our study has some limitations including its retrospective character and the single-center design. However, as a tertiary-care hospital center we were faced with the challenge of rapidly providing medical and surgical services to COVID-19 and non-COVID-19 patients at the same time. Thus, our data offer an important example of “real life” experiences in a rural area (Lower Franconia).

During the COVID-19 pandemic, a cost–benefit analysis for performing elective surgery is necessary to provide sufficient medical, personal and material resources for COVID-19 patients. While it is clear that elective operations in SARS-CoV-2-positive patients should be cancelled or postponed, their delay in non-COVID-19 patients suffering from cancer and other serious conditions can lead to disease progression and impact overall survival. Therefore, instead of recommending unconditional postponement of all “elective” operations, the establishment of COVID-19-free surgical pathways and standardized preoperative SARS-CoV-2 testing can ensure that universally high-quality medical and surgical treatment while minimizing the risk of in-hospital-acquired infections.

## Conclusion

Despite initial studies recommending the postponement of elective surgeries during the COVID-19 pandemic and that non-surgical treatments should be considered for emergency cases, we show that the implementation of a standardized preoperative screening program ensures high-quality surgery for both COVID-19 and non-COVID-19 patients while minimizing infection risk for healthcare workers and potential in-hospital transmission.

## Data Availability

Institutional database. Therefore, restrictions to availability apply due to data protection regulations. Anonymized data are, however, available from the corresponding author on reasonable request.

## Abbreviations

BMI: Body mass index
COVID-19: Coronavirus disease 2019
COPD: Chronic obstructive pulmonary disease
CT: Computer tomography
ICU: Intensive care unit
PCR: Polymerase chain reaction
RT-PCR: Real-time reverse transcriptase polymerase chain reaction
SARS-CoV-2: Severe acute respiratory syndrome coronavirus 2
SD: Standard deviation
